# Patient awareness and renal outcomes in contrast-induced nephropathy: A questionnaire-based analysis

**DOI:** 10.6026/973206300221934

**Published:** 2026-04-30

**Authors:** Ramya Sampathkumar, Zainab Haider Khan, Mahavir Mundra

**Affiliations:** 1 Department of Internal Medicine, Blackpool Teaching Hospitals NHS Foundation Trust, Lancashire, United Kingdom; 2 Department of Medicine, Avalon University School of Medicine, Willemstad Curaçao; 3 Department of Biochemistry, NKPSIMS and Lata Mangeshkar Hospital, Nagpur, India

**Keywords:** Contrast-induced nephropathy (CIN), estimated glomerular filtration rate (eGFR), nephrotoxicity

## Abstract

Patient awareness of contrast-induced nephropathy (CIN) remains poorly characterized despite its clinical relevance and established preventive strategies. This cross-sectional study surveyed 300 adults undergoing contrast-enhanced imaging and retrospectively evaluated renal outcomes to examine whether awareness and perceptions of CIN were associated with post-contrast renal-function decline. Among 270 respondents (90% response rate), the mean awareness score was 4.1 ± 2.3, 6.7% developed CIN and mean eGFR decline was 4.2 ± 3.1 mL/min/1.73 m^2^. Higher awareness scores were significantly associated with lower CIN incidence (4.1% vs. 9.3%, p = 0.02) and independently predicted reduced CIN risk after adjustment for baseline eGFR and contrast volume (OR 0.48, p = 0.03). Thus, we show that patient awareness may represent an actionable dimension of renal-risk mitigation and support integration of structured education into contrast-consent pathways.

## Background:

Contrast-induced nephropathy is the third leading cause of iatrogenic acute kidney injury. The commonest cause is hypo perfusion of the kidneys causing either prerenal injury or acute tubular necrosis [1]. Contrast-Induced Nephropathy (CIN), also known as contrast-induced acute kidney injury (CI-AKI), is a common complication of exposure to iodinated contrast media (ICM) in patients who have chronic kidney disease (CKD), diabetes, advanced age or multiple exposures to ICM [2]. The occurrence of CIN is low in the general population but disproportionately higher among those at risk; as such, it is associated with prolonged hospital stays and higher morbidity rates [3]. In addition to recent advancements in CIN prevention that emphasize risk stratification, adequate hydration, minimization of contrast use and medication review, much variability still exists among clinicians' use and communication of risk, as well as how they manage their patients' contrast exposure peri-procedurally (before, during and after the procedure) [4]. Although clinician knowledge gaps have been documented, little research has focused on the extent to which patients are aware of the risk of CIN and ways to prevent it [5]. Additionally, it is likely that a patient's understanding of the risk of renal injury will affect their adherence to recommendations for hydration pre-procedure, their ability to accurately communicate their renal history and their active participation in the informed consent process [6]. Further, there is limited published evidence evaluating the impact of patient-level awareness on renal outcomes after contrast exposure [7]. Therefore, it is of interest to assess patient awareness of and perceptions regarding CIN and to assess whether there is a correlation between patient awareness and renal function outcomes post-contrast.

## Materials and Methods:

During a period of 6 months at a single tertiary teaching hospital, a cross-sectional observational study evaluated the knowledge level of patients undergoing either contrast-enhanced imaging or interventional radiological procedures. All study participants were ≥ 18 years and were awake and alert enough to give anything but informed consent. However, patients who had a history of acute kidney injury before the study began or patients with end-stage renal disease (ESRD) were not included in this study. Participants in this study were asked to complete a validated questionnaire which assessed the level of awareness of CIN that included knowledge of CIN definitions, risk factors for CIN, prevention methods for CIN and perception of worry or hesitance in regards to CIN and renal complications due to contrast administration. The validated questionnaire was determined to have good internal consistency (Cronbach's alpha of 0.82). Baseline and post- procedure laboratory values, including serum creatinine and estimated glomerular filtration rate (eGFR), were obtained from electronic medical records. The definition of CIN pertains to an increase of at least 25% from baseline serum creatinine value or an increase of at least 0.5 mg/dL from baseline serum creatinine value that developed within 72 hours following contrast exposure. Descriptive statistics were utilized for summarization of participant baseline characteristics and scores from the awareness questionnaire. Chi-square analysis tools were used to analyze the association between categorical variables, independent t-tests were used to compare mean differences in renal parameters across the two groups and Pearson's correlation coefficient was used to analyze the relationship between awareness questionnaire scores and renal parameters. A multivariate logistic regression was performed to determine independent risk factors associated with the likelihood of developing CIN after controlling for participant age, gender, baseline eGFR, diabetes status and volume of contrast administered. Institutional Review Board (IRB) approval was obtained for this research study and all study subjects provided written informed consent prior to participating in this research study.

## Results:

A total of 300 patients were invited to participate and 270 completed the questionnaire, yielding a response rate of 90%. The mean age of participants was 59.3 ± 12.6 years and 52% were male. Most patients were aged 41-60 years (47.4%), followed by those older than 60 years (35.6%). Computed tomography constituted 50% of procedures, while angiography and interventional procedures accounted for 26.7% and 23.3%, respectively. The mean awareness/perception score was 4.1 ± 2.3 on a 10-point scale. Low awareness (0-3) was observed in 31.1% of participants, whereas only 22.2% demonstrated high awareness (7-10). Knowledge regarding CIN definition, risk factors and preventive strategies was limited. The mean post-contrast eGFR decline was 4.2 ± 3.1 mL/min/1.73 m^2^. Eighteen patients (6.7%) met the creatinine-rise threshold consistent with CIN. Renal-function decline and CIN incidence varied across awareness categories. Correlation analysis demonstrated a negative association between awareness score and CIN occurrence. Multivariate regression identified awareness score, baseline eGFR and contrast volume as significant predictors of CIN. Table 1 (see PDF) shows that 52% of participants were male and 47.4% were aged 41-60 years. CT scan was the most frequent procedure (50%), followed by angiography (26.7%) and interventional procedures (23.3%). Table 2 (see PDF) indicates that 31.1% of participants had low awareness, 46.7% had moderate awareness and only 22.2% demonstrated high awareness. The overall mean awareness score was 4.1 ± 2.3, reflecting limited patient knowledge. Table 3 (see PDF) demonstrates that only 34% correctly identified CIN, 29% recognized high-risk conditions and 25% knew preventive measures. Concern regarding contrast use was reported by 28% and 21% were willing to inquire about renal risk. Table 4 (see PDF) compares renal outcomes across awareness categories and shows that mean eGFR decline decreased from 5.1 mL/min in the low-awareness group to 2.8 mL/min in the high-awareness group. CIN incidence was highest in the low-awareness group (9.5%) and lowest in the high-awareness group (5.0%), with statistical significance (p = 0.02). Table 5 (see PDF) depicts a negative correlation between awareness score and CIN occurrence (r = -0.21). Baseline eGFR demonstrated a stronger negative correlation with CIN (r = -0.35), while contrast volume showed a positive correlation (r = 0.28). Table 6 (see PDF) highlights that awareness score independently predicted lower CIN risk (OR 0.48, p = 0.03) after adjustment. Baseline eGFR and contrast volume were also significant predictors, whereas diabetes was not statistically significant.

## Discussion:

This research assessed how conscious patients were about contrast-associated nephropathy (CIN) and how well patient's experienced bad results from contrast-enhanced imaging (CEI) related to their level of awareness [8]. Of the total participants, only 24% (4.1) had some degree of knowledge about CIN, including the definition of CIN, risk factors and how to prevent CIN [9]. But higher levels of awareness were strongly associated with improved renal function (decreased decline in eGFR and lower frequency of CIN) after realising that awareness is still an independent predictor of the development of CIN (after accounting for the baseline renal function and volume of contrast) [10]. These results indicate that awareness is a clinically meaningful factor that may be used to improve the mitigation of renal risk [11]. In patients at high risk for developing contrast-associated acute kidney injury today, the prevalence of contrast-associated acute kidney injury is still present, especially in patients with low baseline renal function, diabetes and high volumes of contrast [12]. The risk of developing CIN due to the use of contrast is supported by the current literature, which indicates that, as with reducing the risk of developing CIN, awareness further validated the independent association that CIN development is dependent on both baseline renal function and the total volume of contrast [13]. While this study confirms the established independent relationship between both baseline renal function and the total volume of contrast as associated factors with developing CIN, it adds the awareness behavioural variable to the traditional model of risk factor identification for predicting occurrences of CIN [14]. The decrease in the mean decline of the eGFR by increasing levels of awareness categories demonstrates the potential impact of behaviour in protecting the kidney in a clinical setting. Increased levels of awareness are associated with better adherence to hydration recommendations, better documentation of renal histories and greater engagement in the process of being informed [15]. Previous studies indicate that patients who become involved will follow instructions more closely and accurately disclose their risk [4, 5]. The behaviours associated with increased awareness will indirectly help with improving renal outcomes by enabling patients and their healthcare providers to better identify risk and optimise their prevention methods [16]. The results of this study highlighted that only 33% of the participants were aware of the term CIN and fewer were aware of how to prevent CIN, suggesting that the current clinical and practice approaches to communicate the risks to renal health and risks associated with the use of contrast agents may not effectively educate patients on the topic [6, 7]. Although improvements in technology and the development of standard practice protocols have led to a lower overall incidence rate for CIN, education is not consistently integrated into the curation of imaging procedures; therefore, education should be provided as a supplement to the existing procedures to maintain baseline renal health [17]. Importantly, the study also showed that the measure of awareness remained an independent predictor of the risk of developing CIN (even after accounting for the best predictors) [18]. Thus, while awareness is an indicator of a patient's baseline health status, it has the potential to be treated as an easily manipulated element of patient-centred care [19]. There is a need to better document patient-reported metrics regarding their level of awareness about the risks associated with contrast procedures throughout the patient education process; thus, making it a key point in promoting shared decision-making and compliance after the procedure [20]. The current study provides evidence that level of patient awareness is not only an educational outcome but that it is positively correlated with measurable outcomes of renal safety. The authors acknowledge several limitations of this study. Due to the cross-sectional study design, no definitive conclusions regarding causation can be made based on the results of this study [21].

## Conclusion:

Low patient awareness of contrast-induced nephropathy is common and independently associated with higher risk of post-contrast renal dysfunction. Awareness score predicted CIN occurrence even after adjustment for baseline eGFR and contrast volume, highlighting its potential role as a modifiable safety factor. Thus, integrating structured patient education into contrast-consent and peri-procedural workflows will strengthen renal-risk mitigation strategies.
